# Cognitive Profiling Related to Cerebral Amyloid Beta Burden Using Machine Learning Approaches

**DOI:** 10.3389/fnagi.2019.00095

**Published:** 2019-04-26

**Authors:** Hyunwoong Ko, Jung-Joon Ihm, Hong-Gee Kim, for the Alzheimer’s Disease Neuroimaging Initiative

**Affiliations:** ^1^Interdisciplinary Program in Cognitive Science, Seoul National University, Seoul, South Korea; ^2^Biomedical Knowledge Engineering Laboratory, School of Dentistry, Seoul National University, Seoul, South Korea; ^3^School of Dentistry, Seoul National University, Seoul, South Korea

**Keywords:** amyloid beta deposition, neuropsychological assessment, machine learning, cognitive profiling, Alzheimer’s disease

## Abstract

**Background:** Cerebral amyloid beta (Aβ) is a hallmark of Alzheimer’s disease (AD). Aβ can be detected *in vivo* with amyloid imaging or cerebrospinal fluid assessments. However, these technologies can be both expensive and invasive, and their accessibility is limited in many clinical settings. Hence the current study aims to identify multivariate cost-efficient markers for Aβ positivity among non-demented individuals using machine learning (ML) approaches.

**Methods:** The relationship between cost-efficient candidate markers and Aβ status was examined by analyzing 762 participants from the Alzheimer’s Disease Neuroimaging Initiative-2 cohort at baseline visit (286 cognitively normal, 332 with mild cognitive impairment, and 144 with AD; mean age 73.2 years, range 55–90). Demographic variables (age, gender, education, and APOE status) and neuropsychological test scores were used as predictors in an ML algorithm. Cerebral Aβ burden and Aβ positivity were measured using ^18^F-florbetapir positron emission tomography images. The adaptive least absolute shrinkage and selection operator (LASSO) ML algorithm was implemented to identify cognitive performance and demographic variables and distinguish individuals from the population at high risk for cerebral Aβ burden. For generalizability, results were further checked by randomly dividing the data into training sets and test sets and checking predictive performances by 10-fold cross-validation.

**Results:** Out of neuropsychological predictors, visuospatial ability and episodic memory test results were consistently significant predictors for Aβ positivity across subgroups with demographic variables and other cognitive measures considered. The adaptive LASSO model using out-of-sample classification could distinguish abnormal levels of Aβ. The area under the curve of the receiver operating characteristic curve was 0.754 in the mild change group, 0.803 in the moderate change group, and 0.864 in the severe change group, respectively.

**Conclusion:** Our results showed that the cost-efficient neuropsychological model with demographics could predict Aβ positivity, suggesting a potential surrogate method for detecting Aβ deposition non-invasively with clinical utility. More specifically, it could be a very brief screening tool in various settings to recruit participants with potential biomarker evidence of AD brain pathology. These identified individuals would be valuable participants in secondary prevention trials aimed at detecting an anti-amyloid drug effect in the non-demented population.

## Introduction

Alzheimer’s disease (AD) is the most common cause of dementia, contributing to about 70% of dementia cases (Plassman et al., 2007). Amyloid beta (Aβ) deposition is a hallmark of AD and begins to accumulate 10–20 years before the clinical onset of AD (Jack et al., 2013). Detection of cerebral Aβ deposition at the presymptomatic stage of AD is very essential, because this intervention makes it possible to identify individuals who would benefit the most from anti-amyloid therapies (Chételat et al., 2010, 2012). Currently, cerebral Aβ deposition can be detected *in vivo* using positron emission tomography (PET) imaging with an Aβ-binding ligand or cerebrospinal fluid (CSF) analysis. However, these processes have several limitations. Using amyloid PET is expensive, and it is not commonly available except in specialized medical center hospitals (e.g., tertiary hospitals). Additionally, it increases exposure to radiation. As for CSF analysis, its use is limited because of the necessity for an invasive lumbar puncture, is labor intensive, and has poor interlaboratory reliability (Mattsson and Zetterberg, 2009). Therefore, developing a new method that is less invasive, less expensive, and accessible in all hospitals could facilitate more effective screening for Aβ deposition. Even if traditional procedures cannot be substituted completely, techniques to help detect Aβ deposition properly should be considered.

Neuropsychological assessment that includes a sensitive and cost-effective clinical measure for evaluating AD could be used to screen for individuals in the preclinical AD phase among cognitively normal (CN) adults and those with mild cognitive impairment (MCI). There is clear value in applying neuropsychological assessment to screen individuals at high risk of developing AD pathology; however, only few studies have shown an association between amyloid deposition and specific cognitive performance among CN participants (Rentz et al., 2010; Sperling et al., 2013; Loewenstein et al., 2016; Schindler et al., 2017). Moreover, there has been little attempt to detect cerebral amyloid deposition using neuropsychological test performance as predictors or to compare the cognitive performance between individuals with high and low levels of Aβ deposition. It is, however, still unclear what specific cognitive performance reflects AD-specific neuropathology. Although many researchers have focused on identifying subtle cognitive changes at presymptomatic stages, there is no consensus to date on a cognitive profile among individuals with Aβ deposition.

Up until now, state-of-the-art machine learning (ML) approaches have rarely been used to detect cerebral Aβ status based on cognitive performance. A previous study used several neuropsychological variables based on ML to distinguish AD from other causes for cognitive impairment but did not look at Aβ status (Gurevich et al., 2017). Most of the studies using an ML algorithm have focused on diagnosis of disease or disease progression based on AD-specific biomarkers, such as volumetric brain measure, cortical thickness, and blood proteins (Moradi et al., 2015; Salvatore et al., 2015; Casanova et al., 2016). Unlike conventional statistical models, ML methods can elucidate multivariate patterns of data, especially useful for highly dimensional and complex data. Furthermore, ML approaches are more effective in minimizing Type I and II errors than univariate statistical methods (Hastie et al., 2009). Given that variables of cognitive function are intricately intertwined, applying ML methods can be helpful in investigating specific patterns of a cognitive profile related to abnormal Aβ deposition.

The goal of the present study was to identify multivariate neuropsychological tests combined with demographic measures, such as age, gender, education, and apolipoprotein E (APOE) 𝜀4 status, using ML algorithm that distinguishes individuals with abnormal levels of cortical Aβ deposition measured by PET in the Alzheimer’s Disease Neuroimaging Initiative (ADNI) sample. The sample includes participants who are CN, have a significant memory concern (SMC), early MCI (EMCI), late MCI (LMCI), or dementia from AD. We also aimed to compare the predictability of the model respectively, based on specific cognitive profiling with variable demographics among several groups of participants within the AD spectrum.

## Materials and Methods

### Ethics Statement

In this study, we used participant data from the ADNI, a multicenter project with approximately 50 medical centers and university sites across the United States and Canada (Petersen et al., 2010). The ADNI was launched in 2003 as a public–private partnership led by Principal Investigator Michael W. Weiner, MD. Its primary goal has been to test whether serial magnetic resonance imaging, PET, other biological markers, and clinical and neuropsychological assessment can be combined to measure the progression of patients with MCI and early AD. Participants were between 55 and 90 years old, and were able to undergo all assessment procedures, and consent to participate in longitudinal follow-up. Written informed consent was obtained from all participants and the study was conducted after prior Institutional Review Board approval was obtained at each participating institution.

### Participants

Cognitively normal participants were the control group in the ADNI study and showed no significant clinical symptoms, including depression, MCI, or dementia. Participants with SMC scored within the normal range for cognitive function but reported concerns about their memory. Participants with EMCI and LMCI reported an SMC either autonomously or via an informant or clinician. However, activities of daily living were preserved, other cognitive domains showed no significant impairment, and no signs of dementia existed. The degree of MCI (early or late) was determined using the Wechsler Memory Scale Logical Memory II (Wechsler, 1984). Participants with AD met the National Institute of Neurological and Communicative Disorders and Stroke Alzheimer’s Disease and Related Disorders Association criteria for probable AD (McKhann et al., 1984; Dubois et al., 2007). A detailed description of the inclusion/exclusion criteria can be found at http://adni.loni.usc.edu/.

Data were downloaded from the ADNI database and included all subjects recruited in the ADNI-2 with complete baseline data available for cognitive assessment, APOE genotype processing and PET Aβ quantitation. Our study sample included 762 subjects (183 control subjects, 103 with SMC, 332 with MCI (175 with EMCI and 157 with LMCI), and 144 with AD) who were recruited between 2011 and 2013, each of whom had a baseline APOE genotype and ^18^F-florbetapir session.

In this study, subgroups were divided into overlapped clinical condition to consider ecological validation. Given the heterogeneity of the clinical spectrum of AD, the clinical standards to determine who was within the disease spectrum cannot always be clear without an accurate identification of the AD biomarkers in the screening tests in clinics or clinical trials. Accordingly, it seemed plausible that dividing the group based on the above properties would reflect the clinical utility of predicting Aβ in various clinical groups. Three groups were specified: mild change group (CN + SMC + EMCI), moderate change group (SMC + EMCI + LMCI), and severe change group(EMCI + LMCI + AD).

### Amyloid PET Data

Baseline Aβ deposition was visualized using florbetapir-PET. Semi-quantitative PET results were retrieved from the latest available dataset (“UCBERKELEYAV45_11_14_17.csv”). The methods for PET acquisition and analysis are described in more detail elsewhere (Landau et al., 2012; Landau et al., 2013). Florbetapir images consisted of 4 × 5 min frames acquired 50–70 min after injection. Images were realigned, averaged, resliced to a common voxel size (1.5 mm), and smoothed to a common resolution of 8 mm in full width at half-maximum (Joshi et al., 2009). Structural T1-weighted images acquired concurrently with the baseline florbetapir images were used as a structural template to define the cortical regions of interest (ROIs), and the reference regions in native space for each subject, using FreeSurfer software (version 5.3.0^1^). Baseline florbetapir scans for each participant were co-registered to baseline structural T1-weighted images. Images were subsequently used to extract weighted cortical retention indices, standardized uptake value (SUV) from gray matter within four cortical ROIs (frontal, anterior/posterior cingulate, lateral parietal, and lateral temporal) that were averaged to generate a mean cortical SUV as described in greater detail online^2^. Cortical SUV ratios (SUVR) were obtained by normalizing cortical SUV with the mean uptake in the whole cerebellum reference region. Participants were classified as cerebral Aβ positive if the florbetapir SUV ratio was greater than 1.1.

### Neuropsychological Assessment

The following measurements were considered: Mini-Mental State Examination (MMSE; Folstein et al., 1975); Alzheimer’s Disease Assessment Scale (ADAS-Cog 13) (Mohs et al., 1997); Montreal Cognitive Assessment (MoCA; Nasreddine et al., 2005); Rey Auditory Verbal Learning Test (AVLT; Rey, 1964); Logical Memory (LM; Wechsler, 1984); Clock Drawing Task (Kaplan, 1983); Trail Making Test (Reitan and Wolfson, 1985); Category Fluency (Morris et al., 1989); Boston Naming Test (BNT; Kaplan et al., 1983); and American National Adults Reading Test (ANART; Grober et al., 1991).

### Statistical Analyses

Groups were first compared using a conventional approach. Demographics were compared between groups using *t*-test. Cognitive variables were compared between groups adjusted for age gender, and education. χ^2^ tests were used to compare dichotomous variables.

Next, to identify multivariate cognitive and demographic profiles that are accurately distinguished from Aβ positivity for participants, the adaptive least absolute shrinkage and selection operator (LASSO) ML algorithm were applied to the dataset (Zou, 2006). The adaptive LASSO, which is a penalized regression method (Tikhonov, 1943), is a popular technique for simultaneous estimation and consistent variable selection (Zou, 2006). With the adaptive LASSO implemented, the regression coefficients of unimportant variables shrank to 0. In that regard, adaptive LASSO algorithm provided interpretable results related to abnormal levels of cerebral Aβ status. In adaptive LASSO, the purpose was to minimize the sum of the square error, but within a constraint (1). The adaptive LASSO estimates are defined as

(1) argminβ||y-∑j=1pxj βj||2+λ∑j=1pwj|βj|

where λ was a shrinkage parameter that controlled the strength of the constraint and **w** was a known weights vector. When λ was close to 0, adaptive LASSO would produce similar estimates as the ordinary least squares method. On the other hand, when λ was large, estimates approached 0 and were removed from the fitted model.

Ten-fold cross-validation was applied during the variable selection process to evaluate the generalizability of the adaptive LASSO model. The data was randomly split into a training set (66.3% of the data) and a test set (33.4% of the data), the adaptive LASSO model was fitted using the training set, and classifications were separately made on the test and training datasets. The optimal parameter, lambda, was determined across 1,000 iterations of 10-fold CV to minimize the deviance of the model. Then, predictions were made on the test set based on the adaptive LASSO model trained in the training set. The area under the curve (AUC) of the receiver operating characteristic (ROC) curve was used as an index of predictability performance. Analyses were performed using R, version 3.4.3^3^.

## Results

### Subject Characteristics

Demographic data for all participants are presented in Table 1. Of the 762 study participants, 350 (46%) were APOE 𝜀4 carriers, and 418 (45%) were Aβ positive (Aβ+).

**Table 1:** Participants characteristics.

Characteristics	All participants
No. of study participants	762
Age, years	72.3 (7.2)
No. of females (%)	363 (48%)
Education, year	16.3 (2.6)
No. of APOE 𝜀4 carriers (%)^a^	350 (46%)
Aβ positivity (%)	418 (45%)
CN, no. (%)	183 (24%)
SMC, no. (%)	103 (13.5)
EMCI, no. (%)	175 (23%)
LMCI, no. (%)	157 (20.6%)
AD, no. (%)	144 (18.9%)

*APOE 𝜀4, apolipoprotein; Aβ, amyloid-beta; CN, clinically normal; SMC, subjective memory concerns; EMCI, early mild cognitive impairment; LMCI, late mild cognitive impairment; AD, Alzheimer’s disease. Data are presented as mean (SD) unless otherwise indicated. ^*a*^APOE 𝜀4 carriers are the percentage of individuals with at least one APOE 𝜀4 allele.*

Table 2 presents demographic and neuropsychological testing by Aβ status for each analytical group. In all groups, the Aβ+ group was older and had more APOE 𝜀4 carriers than the negative Aβ (Aβ-) group. There were significant differences in education between Aβ+ and Aβ-, except for the moderate change group.

**Table 2:** Demographics and neuropsychological characteristics of data sets for machine learning analyses.

	Mild Change (N = 461)			Moderate Change (N = 435)			Severe Change (N = 476)		
	Aβ +	Aβ -	Sig.	Aβ +	Aβ -	Sig.	Aβ +	Aβ -	Sig.
	(*N* = 183)	(*N* = 278)		(*N* = 232)	(*N* = 203)		(*N* = 322)	(*N* = 154)	
Age	74.7 (6.7)	71.8 (6.4)	**0.000**	73.5 (6.8)	71.1 (7.1)	**0.000**	73.9 (7.5)	71.3 (8.1)	**0.001**
Education	16.0 (2.7)	16.7 (2.4)	**0.003**	16.3 (2.8)	16.6 (2.4)	0.162	16.0 (2.7)	16.5 (2.4)	**0.044**
Gender: female	98 (46%)	131 (47%)	0.177	114 (49%)	95 (47%)	0.626	144 (45%)	65 (42%)	0.605
APOE 𝜀4: positive	99 (54%)	63 (23%)	**0.000**	156 (67%)	45 (22%)	**0.000**	233 (72%)	89 (59%)	**0.000**
LM Immediate Recall	12.5 (3.3)	13.4 (3.1)	**0.017**	9.3 (4.0)	11.6 (3.5)	**0.000**	6.7 (3.9)	9.7 (3.3)	**0.000**
LM Delayed Recall	11 (3.2)	12.2 (3.3)	**0.000**	6.8 (4.3)	9.7 (3.7)	**0.000**	4.1 (3.7)	7.3 (3.1)	**0.000**
AVLT Total	40.9 (10.3)	44.8 (11.1)	**0.005**	35.5 (10.7)	41.9 (11.6)	**0.000**	29.2 (10.8)	38.2 (12.2)	**0.000**
AVLT List B	4.5 (1.9)	5.3 (2.0)	**0.004**	4.1 (1.8)	4.9 (1.9)	**0.000**	3.5 (1.7)	4.6 (1.9)	**0.000**
AVLT Delayed Recall	5.9 (3.9)	7.4 (4.2)	**0.003**	4.0 (3.9)	6.4 (4.3)	**0.000**	2.3 (3.2)	5.3 (4.3)	**0.000**
AVLT Recognition	12.1 (2.6)	12.6 (2.6)	0.125	10.8 (3.3)	12.2 (2.8)	**0.000**	8.9 (4.1)	11.4 (3.2)	**0.000**
CLOCK Drawing	4.5 (0.7)	4.7 (0.6)	0.331	4.4 (0.9)	4.6 (0.7)	**0.045**	3.9 (1.3)	4.4 (0.9)	**0.000**
CLOCK Copy	4.8 (0.5)	4.8 (0.4)	0.253	4.7 (0.7)	4.8 (0.5)	**0.018**	4.5 (0.9)	4.7 (0.6)	**0.005**
Category Fluency (Animals)	19.3 (5.6)	20.5 (5.3)	0.306	17.5 (5.4)	19.5 (4.9)	**0.002**	15 (5.7)	18.5 (5.3)	**0.000**
Boston Naming Test	27.4 (2.6)	28.1 (2.5)	0.070	26.3 (3.6)	27.8 (2.5)	**0.000**	24.3 (5.2)	27 (3.5)	**0.000**
TMT A (seconds)	37.1 (14.8)	33.1 (11.3)	**0.028**	40.3 (18.6)	35.2 (13.5)	**0.012**	49.5 (28.6)	37.1 (14.1)	**0.000**
TMT B (seconds)	98.3 (52.6)	82.9 (44)	0.069	114.5 (68)	90.6 (47.4)	**0.002**	138.7 (89.7)	98.9 (58)	**0.000**
ANART (# of Errors)	11.1 (8.6)	9.8 (7.4)	0.806	12.0 (9.5)	11.3 (8.0)	0.961	14.0 (9.8)	12.1 (8.7)	0.301
MOCA	23.6 (2.9)	24.1 (1.9)	0.142	23.2 (3.0)	23.8 (2.0)	0.070	21.3 (4.2)	23.2 (2.8)	**0.000**
ADAS-Cog 13	12.0 (5.4)	9.6 (4.9)	**0.000**	16.4 (7.4)	11.4 (5.7)	**0.000**	22.9 (10.4)	14.4 (7.7)	**0.000**
MMSE	28.6 (1.5)	28.9 (1.4)	0.100	27.8 (1.8)	28.7 (1.4)	**0.000**	25.8 (3.0)	27.9 (2.3)	**0.000**

*ADAS-Cog 13, 13 item version of the cognitive subscale of the Alzheimer’s Disease Assessment Scale-Cog; ANART, American National Adults Reading Test; APOE 𝜀4, apolipoprotein epsilon 4 allele carrier; AVLT, Rey Auditory Verbal Learning Test; LM, logical memory; MMSE, mini-mental status examination; MOCA, montreal cognitive assessment; TMT, Trail Making Test. Count (%) or mean (standard deviation). Comparisons for cognitive performances were adjusted for age, gender and education. *P*-values are from *F*-tests and Pearson chi-square tests. Significant p-values are highlighted in bold.*

For the neuropsychological assessment, the differences between participants who were Aβ+ and Aβ- were greater than differences in participants with pathological changes.

### Adaptive LASSO Results

**FIGURE 1:**
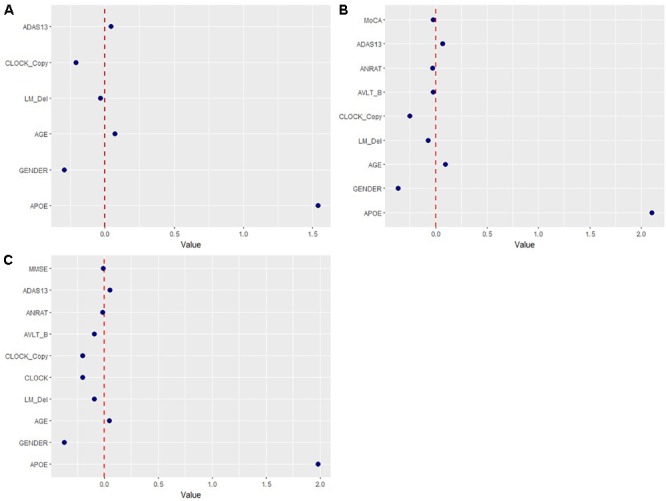
Multivariate patterns of demographic information and cognitive measures predicting amyloid positivity in mild **(A)**, moderate **(B)**, and severe group **(C)**. ADAS13, Alzheimer’s disease assessment scale; APOE, ApoE 𝜀4 positivity; AVLT, Rey auditory verbal learning test; AVLT _B, Rey auditory verbal learning test list B; ANRAT; American national adults reading test; BNT, Boston naming test, CLOCK, clock drawing task, CLOCK_Copy; clock drawing task copy; LM_del, logical memory delayed recall; MMSE, mini-mental state examination; MoCA, Montreal cognitive assessment scale.

The predictability of demographic (age, gender, and APOE 𝜀4 status) and neuropsychological data of participants was assessed for ability to predict cerebral amyloid positivity. Figure 1 shows the multivariate profiles for cerebral Aβ positivity, revealed by ML algorithm. First, Aβ positivity was more prevalent in participants who were older, female, APOE 𝜀4 carriers, and showed poor cognitive performance in several measures in the mild change group (worse delayed recall, clock drawing, and ADAS-Cog 13). In the moderate change group, the demographics were like those in the mild change group. Several additional cognitive performance variables were added to the results for the mild change group (worse delayed recall, clock drawing, AVLT list B, BNT, ANART, ADAS-Cog 13, and MMSE score). In the severe change group, demographic variables and cognitive performances variables (LM delayed recall, clock drawing, AVLT list B, ANART, ADAS-Cog 13, and MMSE) also predicted Aβ positivity.

**FIGURE 2:**
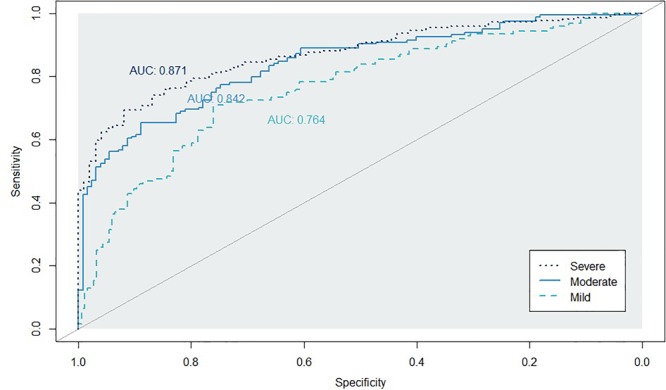
Classification accuracy as indexed by the receiver-operating characteristic (ROC) curves and their area under the curve (AUC) on the training set. Mild, mild change group (CN+SMC+EMCI); Moderate, moderate change group (SMC+EMCI+LMCI); Severe, severe change group (EMIC+LMCI+AD).

Figure 2, 3 show the ROC curve and its AUC for the classification of Aβ positivity. For the mild change group, the AUC was 0.764 for the training set and 0.754 for the test set. For the moderate change group, the AUC was 0.840 and 0.811 for the training and test sets, respectively. For the severe change group, the AUC was 0.871 and 0.864 for the training and test sets, respectively.

**FIGURE 3:**
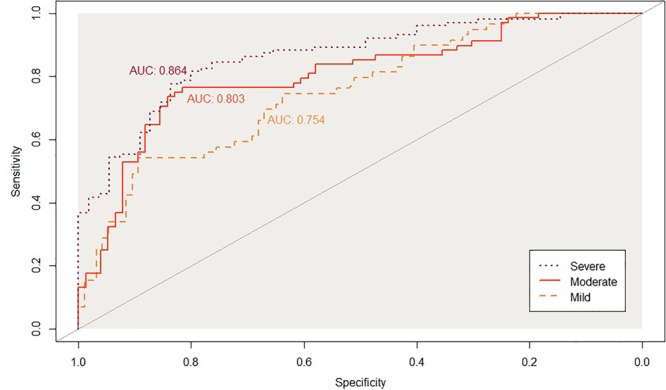
Classification accuracy as indexed by the receiver-operating characteristic (ROC) curves and their area under the curve (AUC) on the testinkg set. Mild, mild change group (CN+SMC+EMCI); Moderate, moderate change group (SMC+EMCI+LMCI); Severe, severe change group (EMIC+LMCI+AD).

## Discussion

This study confirmed that multivariate profiles of neuropsychological assessment with demographic measures could efficiently predict Aβ positivity using an ML method. The adaptive LASSO algorithm selected a subset of variables that were most predictive of Aβ positivity, whereas the estimates of other variables were 0 due to its penalized procedure. Although some studies have reported high predictive accuracy for Aβ positivity based on ML methods, these studies were based on blood biomarkers or combined with neuropsychological tests (Burnham et al., 2014; Haghighi et al., 2015). To our knowledge, this is one of the first studies that demonstrates relative profile predicting cerebral amyloid status based on an ML algorithm using only demographic and neuropsychological measures.

Current findings show that neuropsychological test performance and demographics can predict Aβ positivity with about 80% predictability in the non-demented population. Given these results, specific neuropsychological measures have implications for early detection of neuropathological biomarkers in AD without invasive methods such as PET and CSF analysis.

In the mild change group (CN to EMCI), clock drawing, LM delayed recall, and ADAS-Cog 13 are significant predictors for Aβ positivity with demographic measures (age, gender, and APOE 𝜀4 status). The LM test, which consists of two brief stories, is also very sensitive to early episodic memory decline before the clinical onset of AD (Rubin et al., 1998) even before the onset of MCI (Howieson et al., 2008). According to a recent review of studies with preclinical neuroimaging and prospective cohorts, the LM test has the most consistent association with the amyloid level among CN individuals (Mortamais et al., 2017). Moreover, a recent study using the preclinical Alzheimer’s cognitive composite demonstrated that logical memory delayed recall test consistently improved the effect sizes at less than 5 years follow-up in CN participants (Mormino et al., 2017). The ADAS-Cog is considered the gold standard for estimating the effectiveness of anti-dementia treatments (Kueper et al., 2018). Although the ADAS-Cog was developed for use in clinical trials of dementia, a study using ADNI dataset showed results reflecting Aβ-related decline in performance on the ADAS-Cog (Li et al., 2017). In the current study, the Aβ+ with APOE 𝜀4 carriers group shows significantly impaired performance on the ADAS-Cog test but not the MMSE among CN and EMIC participants compared with other groups. The result is consistent with a recent meta-analysis focused on cognitive impairment and high Aβ status in CN individuals (Baker et al., 2017). The general cognitive impairment in Aβ+ CN individuals would indicate that increased Aβ burden is related to disturbed cognitive function even in very early stage of AD. It is interesting that the coefficient of the Clock Drawing Task from penalized regression was higher than the other cognitive measures. Previous studies showed that poor performance of the Clock Drawing Task was related to cortical dysfunction in the bilateral temporoparietal regions (Shon et al., 2013). It has also been found that Aβ+ CN older adults have more amyloid burden as measured by Pittsburgh Compound B PET imaging in these regions compared with Aβ- older adults (Sperling et al., 2009). In that regard, visuospatial impairment in copying task may capture a very early Aβ-related sign.

In the moderate change group, as the degree of the disease progresses, three neuropsychological measures are added for predicting Aβ positivity. The AVLT list B has 15 different words than the AVLT list A, which serves as an interference trial. A previous study showed that intrusion errors from a word-list episodic memory test and APOE 𝜀4 status significantly predicted progression to AD in CN elderly individuals (Bondi et al., 1999). It may be possible that elevated errors on the AVLT list B arise because of deficits in semantic memory storage for learning after the AVLT list A has been learned. A recent study also showed that the AVLT intrusion errors predicted progression from CN to MCI and CN to clinical symptom of dementia (i.e., clinical dementia rating = 1), suggesting that intrusion errors were likely to reflect subtle change during early AD pathology. With respect to the ANART, it is a premorbid intelligence test that is known as a proxy of cognitive reserve (CR), which may explain how some individuals can preserve normal cognitive function despite pathological change, such as cortical atrophy (Stern, 2012). It may be possible that people with high performance on the ANART have higher CR than those with low performance on the ANART. That is, if there is an increase in CR accompanied by impairment in other cognitive domains, it is likely to reflect neuropathology in AD more than low CR, at the same time as compensating for other cognitive function. Indeed, it is consistent with the study that early intellectual enrichment, including educational attainment, is associated with an increase in higher florbetapir-PET uptake in MCI, suggesting a compensatory increase for Aβ burden (Arenaza-Urquijo et al., 2017). The MoCA has been developed as a more challenging test that measures higher-level language, complex visuospatial ability, and executive function to enable the detection of MCI and to address the inability of the MMSE to detect MCI (Nasreddine et al., 2005). The MoCA may have more sensitivity to neuropathological changes in AD compared with the MMSE. In the severe change group, in addition to results of the moderate group, there is one more significant cognitive measure to predict Aβ status. The lower performance on Clock Drawing Test is related to higher possibility of Aβ positivity. Due to changes in the brain as the disease progresses, episodic memory ability may be reduced and the ability to draw from memory impaired. Thus, considering the other damage to episodic memory, including LM and AVLT list B, poor performance on Clock Drawing Test would suggest neuropathological change as a marker of AD.

Across all groups, age, gender, and APOE 𝜀4 status are significant variables to predict Aβ positivity. It is well established that cortical Aβ burden increases with older age and the APOE 𝜀4 genotype (Morris et al., 2010; Jansen et al., 2015). As for gender, a recent study revealed that CN females who had a lower testosterone level were more likely to be Aβ+ than those with a higher level (Lee et al., 2017). It is notable that several cognitive measures show higher predictability for Aβ positivity than age. Considering these findings, it seems that Aβ-related profiles of cognitive measures, although subtle, can detect AD-specific changes before the clinical onset of AD.

Overall, the result of the highly predictive model for the moderate change group suggests that multivariate profiles in cognitive and demographics measures based on ML can be useful for non-demented individuals, including those with SMC and MCI, who are targeted for anti-amyloid therapeutic intervention as a preliminary screening tool before undergoing invasive methods. There are medical benefits in early diagnosis of individuals with AD pathology. The earlier that a diagnosis is determined, the earlier that medical intervention can begin, which can delay cognitive decline and disease progression in individuals at preclinical stages of AD.

In addition to an early intervention, there is a potentially financial benefit of early detection of Aβ positivity. Many people have been impacted by the high cost of amyloid PET. An amyloid PET scan costs approximately $5,000 per person in the United States (O’Bryant et al., 2017). Specifically, current findings can be used to identify those who should undergo amyloid PET imaging for inclusion in clinical trials or anti-amyloid therapy. Considering the increasing ability to distinguish Aβ+ from Aβ-, the availability of this cognitive profile model would result in a significant cost saving for dementia caregivers as well as clinical trials.

**FIGURE 4:**
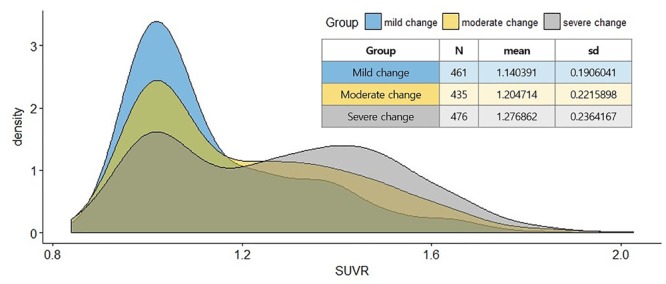
Distributions of the Aβ retention (SUVR) across groups.

Potential limitation of this study exists in the cross-sectional design, and any inference about progression from the asymptomatic to clinical stages must be cautiously examined. Moreover, ADNI is not reflective of the general population. However, it compares multiple cognitive scales related to Aβ burden simultaneously, which estimates the relative effects on Aβ positivity based on several subgroups reflecting actual clinical practice. Considering the change in distribution to Aβ status (SUVR) in Figure 4, the distribution becomes dichotomous as the extent of the disease develops. This bimodal distribution has been previously reported for a study using the ADNI dataset (Ewers et al., 2011). This result would reflect the neuropathological changes in the continuous course of AD, which may suggest a causal relationship between cognitive function and Aβ deposition. Our findings also demonstrate potential benefits in clinical utility for non-demented individuals as well as out-of-sample generalization using an ML algorithm. Further studies are needed to specify the robust relationship between cognitive function and Aβ burden by using longitudinal design. Developing novel assessments to capture subtle cognitive impairment related to Aβ among individuals those who are clinically normal is also a priority.

## Conclusion

In conclusion, our findings demonstrate that multivariate neuropsychological assessment and demographic measures using an ML algorithm might predict abnormal level of Aβ status in the non-demented population. Results also provide useful cognitive markers related to Aβ deposition, suggesting subtle changes in preclinical stage of AD. Application of these findings may help more specific identification of Aβ-related changes in cognition at the early stage of AD than before, which can contribute to the development of precision medicine in the field of AD research and therapy.

## Author Contributions

HK and H-GK designed the study, acquired and interpreted the data, and were major contributors to the writing of the manuscript and critically revising the manuscript for intellectual content. J-JL analyzed the data and helped to draft the manuscript.

## Conflict of Interest Statement

The authors declare that the research was conducted in the absence of any commercial or financial relationships that could be construed as a potential conflict of interest.

## Footnotes

1 http://surfer.nmr.mgh.harvard.edu

2 http://adni.bitbucket.org/docs/UCBERKELEYAV45/UCBERKELEY_AV45_Methods_12.03.15.pdf

3 http://www.r-project.org/
